# The delivery of essential newborn care in conflict settings: A systematic review

**DOI:** 10.3389/fped.2022.937751

**Published:** 2022-11-01

**Authors:** Vinay Kampalath, Sarah MacLean, Abrar AlAbdulhadi, Morgan Congdon

**Affiliations:** ^1^ Division of Emergency Medicine, Department of Pediatrics, Children's Hospital of Philadelphia, Philadelphia, PA, United States; ^2^ Center for Global Health, Children's Hospital of Philadelphia, Philadelphia, PA, United States; ^3^ London School of Hygiene and Tropical Medicine, University of London, London, United Kingdom; ^4^ Perelman School of Medicine, University of Pennsylvania, Philadelphia, PA, United States; ^5^ Division of General Pediatrics, Children's Hospital of Philadelphia, Philadelphia, PA, United States; ^6^ Section of Hospital Medicine, Division of General Pediatrics, Department of Pediatrics, Global Children's Hospital of Philadelphia, Philadelphia, PA, United States

**Keywords:** pediatrics, neonatal, humanitarian, refugee, perinatal mortality, global health, conflict

## Abstract

**Introduction:**

Although progress has been made over the past 30 years to decrease neonatal mortality rates, reductions have been uneven. Globally, the highest neonatal mortality rates are concentrated in countries chronically affected by conflict. Essential newborn care (ENC), which comprises critical therapeutic interventions for every newborn, such as thermal care, initiation of breathing, feeding support, and infection prevention, is an important strategy to decrease neonatal mortality in humanitarian settings. We sought to understand the barriers to and facilitators of ENC delivery in conflict settings.

**Methods:**

We systematically searched Ovid/MEDLINE, Embase, CINAHL, and Cochrane databases using terms related to conflict, newborns, and health care delivery. We also reviewed grey literature from the Healthy Newborn Network and several international non-governmental organization databases. We included original research on conflict-affected populations that primarily focused on ENC delivery. Study characteristics were extracted and descriptively analyzed, and quality assessments were performed.

**Results:**

A total of 1,533 abstracts were screened, and ten publications met the criteria for final full-text review. Several barriers emerged from the reviewed studies and were subdivided by barrier level: patient, staff, facility, and humanitarian setting. Patients faced obstacles related to transportation, cost, and access, and mothers had poor knowledge of newborn danger signs. There were difficulties related to training and retaining staff. Facilities lacked supplies, protocols, and data collection strategies.

**Conclusions:**

Strategies for improved ENC implementation include maternal and provider education and increasing facility readiness through upgrades in infrastructure, guidelines, and health information systems. Community-based approaches may also play a vital role in strengthening ENC.

## Introduction

Recent humanitarian crises in Ethiopia, Afghanistan, Syria, Yemen, and Ukraine have focused international attention on the health impacts of armed conflicts. In these contexts, children are especially vulnerable. In 2021, the United Nations High Commissioner on Refugees estimated that of the unprecedented 84 million forcibly displaced people worldwide, 42% (or 35 million) were children (1). Of all children, newborns are among the most vulnerable; since 2018, at least one million babies have been born into refugee status (1).

Over the last 30 years, global reductions in neonatal mortality (defined as deaths within the first 28 days of life) have not been as dramatic as reductions in mortality among children under five years of age (under-5s) (2). Currently, at least 60 countries are estimated to miss the Sustainable Development Goal of a neonatal mortality rate of under 12 deaths per 1,000 live births by 2030 (3). Geographically, the pattern of neonatal mortality is uneven, with higher neonatal mortality rates occurring in conflict-affected countries (4). Increasingly, global neonatal mortality is concentrated in countries affected by conflict and displacement, such that of the 15 countries with the highest neonatal mortality rates in the world in 2015, 14 were experiencing conflict and displacement at the time (5, 6). For example, while global neonatal deaths account for 38% of all under-5 deaths, in some conflict settings, such as Myanmar and Yemen, that number has increased to 53% (7, 8).

As a result of these disparities, attention has turned to reduce neonatal mortality in humanitarian crises. One such effort is the Newborn Health in Humanitarian Settings: Field Guide, developed by the Inter-Agency Working Group on Reproductive Health in Crises (7). Developed in 2017, the Field Guide provides guidelines designed to reduce neonatal mortality in humanitarian settings. It describes essential newborn care (ENC) as a set of fundamental services for every newborn. ENC includes thermal care, initiation of breathing, feeding support, and infection prevention. ENC also describes several preventive and promotive health actions, including the identification of neonates in need of advanced care, the dispensation of anticipatory guidance to families, and postnatal checks during the first month of life (7). The Field Guide also details interventions for babies born prematurely or with low birth weight, babies with suspected serious bacterial infections, and babies with a history of intrapartum complications. The Field Guide organizes recommendations at three service delivery levels: the household, primary care facility, and hospital. In this systematic review, we sought to understand the barriers to and facilitators of ENC implementation in humanitarian settings, particularly at the facility and hospital levels.

## Methods

The search strategy was designed by all study authors. This review adhered to the Preferred Reporting Items for Systematic Reviews and Meta-Analyses (PRISMA) statement (9). Articles were accepted for screening only if they included search terms from each of three separate concepts: (a) the conflict setting, (b) newborns, and (c) ENC. The full syntax for the systematic search strategy was informed by previous systematic reviews that focused on health in humanitarian crises and is available in Supplementary Appendix S1 (10, 11). Two librarians were consulted to review the syntax.

**Figure 1:**
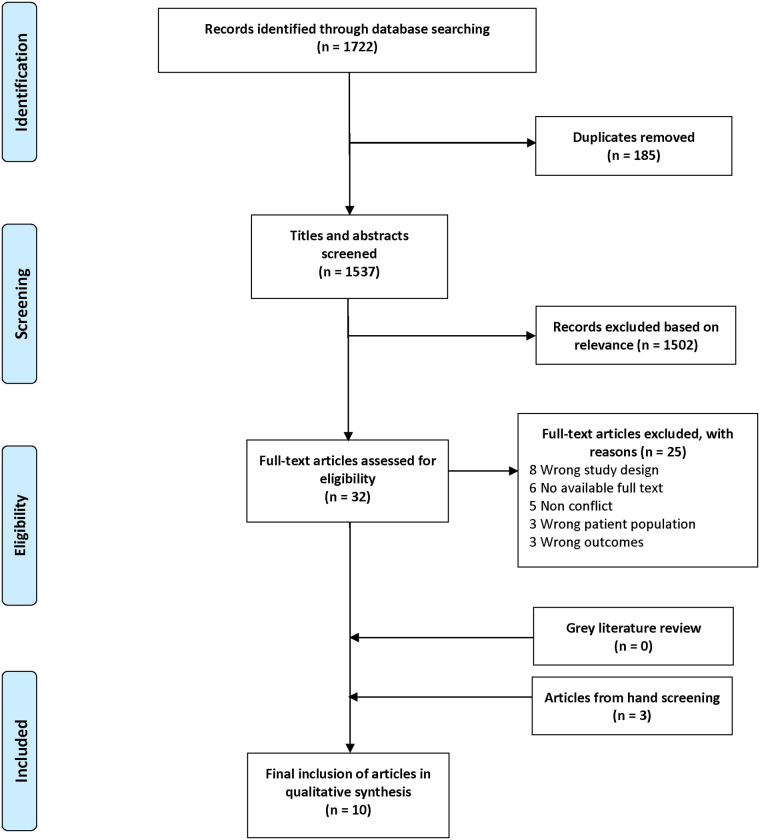
Flowchart of database search results and record screening.

All identified citations were uploaded into Covidence, a systematic review management platform. Duplicates were removed automatically by Covidence and a standard abstract screening process was applied. Two reviewers (AA, SM) independently screened each abstract for relevance to the subject matter. All studies considered eligible for full-text review by both reviewers were automatically included, and disagreements were resolved by consensus with a third reviewer (MC or VK). Each full-text manuscript was assessed for eligibility by two reviewers, and disagreements were again resolved by consensus with a third reviewer. Grey literature was reviewed similarly. Two authors (MC, VK) hand-searched included articles’ reference lists to determine additional eligible articles. The manuscripts deemed eligible for final inclusion underwent data extraction by two reviewers to identify the study design, methods, aims, and findings. The team evaluated data extraction results to achieve consensus. The PRISMA flowchart, which details this search strategy, can be found in Figure 1.

We searched published literature in English from the beginning of November 2017, the date that the Field Guide was published, through September 28, 2021. Peer-reviewed literature was searched using CABI Global Health, Excerpta Medica Database (Embase), Ovid, Cumulative Index to Nursing and Allied Health Literature (CINAHL), and the Cochrane Database of Systematic Reviews. We searched for grey literature in databases from the Healthy Newborn Network, International Rescue Committee, International Committee of the Red Cross, Médecins Sans Frontières (Epicentre and Field Research), Save the Children, and UNICEF. Reference lists from retrieved articles were screened for relevant studies.

For this review, a humanitarian crisis was defined as a circumstance in which civilian mortality was significantly and persistently above a population's baseline, such that there was severe disruption to society, and there was a requirement for support from national, international, and multilateral partners (10, 12). Internationally accepted definitions of refugees and internally displaced people (IDPs) were utilized in referring to affected populations (13).

### Eligibility criteria

Our target population included neonates, defined as infants in the first 28 days of life. We excluded resettled refugee populations. Eligible publications included a description of ENC in a conflict setting, with a description of barriers or facilitators. Our search focused on interventions targeting the newborn, so we excluded literature that focused on the antenatal period. We excluded case reports, editorials, rapid assessments, and non-scientific reports. We applied the same inclusion criteria for indexed grey literature. The complete list of inclusion and exclusion criteria is found in Table 1.

**Table 1:** Inclusion and exclusion criteria for systematic review.

Subject	Inclusion criteria	Exclusion criteria
Populations	- Refugees, IDPs, and other populations affected by conflict - Neonates (defined as infants in the first 28 days of life)	- All other children (e.g., resettled refugees) - Children aged 29 days of life and greater (NB: Articles that discuss infants up to two months of age will be included if they are grouped with neonates aged day 0–28.)
Outcomes	- Delivery of ENC, with a focus on barriers and facilitators	- Any other outcome - ENC components exclusively related to antenatal period
Study designs	- Primary research of any quantitative or qualitative study design from academic and grey literature that describes an ENC intervention and its delivery	- Case series/reports, rapid assessments, field assessments, non-scientific reports
Language	- English	- Any other language
Dates	- Publish dates spanning 1 November 2017 to 28 September 2021 (date that review was conducted), inclusive	- Publish dates 31 October 2017 and earlier

### Quality assessment

The quality of included studies was assessed using the Strengthening the Reporting of Observational Studies in Epidemiology (STROBE) guidelines (14). Each full-text article received quality assessment by two reviewers, and discrepancies were resolved through group consensus. Studies were awarded one point for each item of the 22-point STROBE checklist. The total points awarded to each study were divided by the total possible points to obtain a percentage score. Studies were defined as low, moderate, and high quality with scores of <33%, 33%–66%, and >66%, respectively.

## Results

Ten articles were included for final review. The details of these studies are found in Table 2. Notably, none of the 572 screened grey literature records met the eligibility criteria for final inclusion. The ten articles represent nine countries [Afghanistan, Cameroon, Chad, the Democratic Republic of Congo (DRC), Niger, Somalia, South Sudan, Uganda, and Yemen]. The barriers and facilitators that emerged from this systematic review were subdivided by level (patient, staff, facility, and humanitarian setting). Some factors are cross-cutting and appear at multiple levels.

**Table 2:** Overview of included studies with study characteristics and summaries of key findings.

First author	Study setting and year	Participants and sample size	Summary of findings
Amsalu 2019	Four PHC facilities in Somalia (2016)	253 women, aged 15–49 years old, who delivered at PHC facilities	- The majority of childbirths were attended by skilled health workers, but quality of care varied - ENC interventions were not universally available - There was a low prevalence of handwashing, breastfeeding support, and skin to skin placement, but drying occurred
Amsalu 2020 (Somalia, Chad, Cameroon, Niger)	Healthcare facilities in conflict settings - Somalia (2016–2020), Chad, Cameroon, and Niger (2018–2019)	404 HCWs in conflict settings in Somalia, Chad, Cameroon, and Niger	- After Helping Babies Survive training, providers’ knowledge improved and skills in newborn resuscitation improved (mean score difference +65.1%) - Learned skills were retained at the 18-month follow up - Low-dose, high-frequency trainings were effective in building competence - Lack of equipment made trainings difficult - Cross-functional trainee groups improved team dynamics
Amsalu 2020 (Somalia)	One healthcare facility in Somalia (2016–2018)	690 women, age 15–49 years, who sought childbirth care	- Intervention package (provider education, newborn supply provision, development of newborn registry) was feasible and effective in improving ENC at facility level - Knowledge and skills gained after training were mostly retained at the 18-month follow-up - Some aspects of ENC (handwashing, pre-discharge education) did not improve - Newborns receiving 2+ ENC practices improved from 19.9% at baseline to 94.7% at endline
Atiqzai	Public health facilities with at least 5 births per day on average in Afghanistan (2016)	Managers, SBAs, and mother-baby dyads at 226 public health facilities	- There was no specific pattern in availability of supplies and guidelines for ENC - SBA knowledge of ENC was slightly higher at district hospitals and PHC facilities compared with provincial, regional and specialty hospitals where the majority of facility births occur - There was inadequate knowledge of low-cost, high-impact ENC practices - There were inadequacies in pre-discharge examinations, postpartum counseling, postpartum care, warning about newborn danger signs
Eze	Single-center NICU in Yemen (2017–2018)	976 neonates (<29 days)	- Predictors of neonatal deaths were preterm birth, LBW, and traveling >60 min to NICU - Most common admission diagnoses were complications of prematurity (34.9%), perinatal asphyxia (34.4%), neonatal jaundice (18.8%), and neonatal sepsis (16.1%)
Hynes	Health facilities in DRC (2015–2016)	394 women aged >18 who underwent uncomplicated SVD	- Both the enhanced intervention group (receiving an additional QI methodology to test changes to care) and the control group showed improvements over time following clinical training on BEmONC, ENC, and partograph use - Enhanced intervention group showed a greater rate of change than the control group for AMTSL and ENC and achieved 100% ENC completion at endline
Komakech	Refugee settlements in one district of Uganda (2016)	Women, aged 18–49 years, who had delivered a baby in the last six months	- 57% of mothers breastfed their newborns within an hour, 50.1% cleaned their newborns’ cord appropriately, 12.7% of newborns had a bath >24 h after birth, and 17% of newborns received optimal thermal care - Mothers aged 20–24 were less likely to perform good cord care - Mothers with the least primary education and those who identifed as Catholic were more likely to have initiated breastfeeding early
Mizerero	Public facilities in DRC (2006–2013)	Data on 35,283 deliveries	- No health centres could provide all basic EmONC services - Only 30% of the minimum acceptable number EmONC facilities was met - Resources needed for EmONC and institutional referral were scarce in surveyed health centers
Sami	Health facilities in displacement camps in South Sudan (2016)	343 mother-baby dyads and 13 midwife birth attendants who provided services	- Mothers’ knowledge of danger signs was poor
			- Postnatal monitoring was inconsistently observed, but those babies delivered by SBAs (as opposed to TBAs) were more likely to receive postnatal monitoring
			- Certain practices critical to ENC were less likely to be observed by unskilled birth attendants
			- Service availability and readiness for ENC was low at the facility level (electricity, supplies, staffing not adequately available)
			- Midwives spent most of their time on non-patient-contact activities
Tappis	Yemen (2015–2018)	National and sub-national government authorities, humanitarian agency staff, and HCWs (181 total)	- Service delivery and care-seeking patterns have been impacted by insecurity, reduced availability of health services, and economic downturn - The deterioration of government authority in Yemen has exacerbated humanitarian assistance and made governance difficult - Challenges to service delivery and intervention coverage included insecurity (attacks against healthcare specifically, stress among staff), politicization of aid (national, subnational, and non-state actors), health system capacity, and cost barriers to seeking care - Staff members were incentivized to leave Yemen or move from public to private clinics due to better pay

Abbreviations: AMTSL, active management of the third stage of labor; BEmONC, basic emergency obstetric and newborn care; DRC, Democratic Republic of Congo; ENC, essential newborn care; LBW, low birth weight; PHC, primary health care; NICU, neonatal intensive care unit; QI, quality improvement; SBA, skilled birth attendant; TBA, traditional birth attendant.

**Table 3:** Barriers to ENC delivery and strategies for improvement, by level.

Level	Barriers	Strategies for improvement
Patient	• Insufficient maternal knowledge of newborn danger signs • Transportation barriers (e.g., distance, security checkpoints) • Cost (e.g., referral, medicines)	• Initiatives designed to improve maternal education and build trust within communities • Community-based strategies (e.g., community midwives)
Staff	• Inadequately trained staff • Pay differentials between public sector and international NGOs • Time constraints from administrative duties and splitting time between clinical settings	• Dedicated trainings for HCWs • Mentorship by supervisors • Incentives such as promotion and pay-for-performance
Facility	• Variable service availability or coverage • Inadequate infrastructure and supplies • Lack of interfacility referral mechanisms	• Mother-baby-friendly spaces • Up-to-date clinical guidelines and standard operating procedures (SOPs) • Adoption of neonatal signal functions • Improvements in health information systems
Humanitarian setting	• Insecurity • Donor-driven prioritization • Breakdown in governance • Attacks against healthcare	• “Strategic” governance • Negotiated access to populations in need

### Patient-related factors

There were several patient-level barriers to ENC delivery. A case study in Yemen reported on factors that impeded patient access to facility care, such as geographical constraints, roadside checkpoints, and transportation (15). Yemeni women, who were noted to traditionally prefer home births, would only seek facility-based care when experiencing complications due to difficulties related to access. In a study from the DRC, 80% of women arriving at emergency obstetric and neonatal care (EmONC) facilities traveled by foot, some for up to two days (16). Transportation was also an impediment when newborns required referral for intensive care (17). As Eze, et al*.* reported, over two-thirds of newborns in northwestern Yemen traveled for over an hour for transfer. Tappis, et al*.* reported that other demand-side factors, such as distrust in health providers and reprioritization of needs, contributed to decreased acceptability of certain preventive services, including immunization (15). Two studies also reported that costs related to transportation and medications were distinct barriers for patients (15, 18).

A key element of postnatal ENC includes counseling families on recognizing newborn danger signs, so that they seek care for unwell babies. In several studies, low maternal knowledge of these warning signs, hygienic cord care, and breastfeeding reflected inadequate ENC delivery (19–21). In one survey of four health facilities in Somalia, only 40% of mothers received pre-discharge education on ENC due to the short length of stay after childbirth and inadequate staff training (21). In a descriptive study evaluating newborn care in South Sudan, maternal knowledge of newborn danger signs was suboptimal; only 20% of mothers could identify four or more danger signs (20). In a quasi-experimental study in Somalia that evaluated the introduction of an intervention based on the Field Guide, pre-discharge education for mothers on breastfeeding and newborn danger signs had no significant improvement following the intervention, highlighting the challenge to achieving meaningful gains in maternal education (22).

### Staff-related factors

Some of the variability in implementation of ENC was partly due to variation in staff skill level (21, 23). A study in displacement camps in South Sudan found that newborns delivered by skilled birth attendants (SBAs) were more likely to receive postnatal monitoring compared to those delivered by traditional birth attendants (TBAs) (RR: 1.59, 95% CI: 1.09–2.32) (20). Observations of clinical practice in Afghan public hospitals demonstrated that high-impact, low-cost, and low-technology ENC actions, such as thermal care, delayed cord clamping, and breastfeeding promotion, were not routinely conducted (23). Pre-discharge counseling of mothers was also noted to be frequently omitted or insufficient in many studies (22–24).

Several reviewed records reported educational initiatives that successfully trained health care workers (HCWs) on ENC and improved newborn outcomes. A study in Somalia, for example, assessed the impact of an intervention package consisting of HCW training, distribution of newborn-specific commodities, and the development of a newborn register on three ENC behaviors (skin-to-skin contact, dry cord care, and early breastfeeding). Amsalu, et al*.* demonstrated that after the intervention was implemented, newborns were more likely to receive at least two (OR: 64.5, 95% CI: 15.8, 262.6, *P*-value <0.001) or all three (OR: 220.0, 95% CI: 33.7, 1443.0, *P*-value: <0.001) ENC interventions (22). Another study from the DRC showed that an experimental group receiving a participatory quality improvement initiative designed to improve facility-based maternal and neonatal care reached 100% ENC coverage at the end of the study period, with a greater rate of change in the uptake of ENC compared with the control group (OR: 49.62: 95%, CI: 2.79–888.28) (16). Other studies demonstrated that frequent refresher trainings and cascade training models aided in long-term knowledge retention and created an environment that maximized patient care and staff training, respectively (15, 24).

Retaining qualified HCWs in conflict settings was a recurrent barrier. HCWs reported being overworked. Midwives in Yemen were pulled into primary care practice in the community and had less time to devote to their EmONC activities (15). In South Sudan, midwives only spent 40% of their routine workday in direct patient care; the remaining 60% of their time was spent in non-patient-facing tasks such as documentation, meetings, supervision, and cleaning (20). Furthermore, pay differentials, particularly among international non-governmental organizations, private clinics, and public health centers, contributed to staff turnover. In Yemen, Tappis, et al*.* noted that harmonizing cross-sector financial and non-financial incentives would contribute to maintaining levels of providers for adequate care across the health system (15).

### Facility-related factors

In the articles surveyed for this systematic review, many facilities had variable or low availability of ENC services (18, 20, 21). In some cases, not all facilities offered newborn services. In other settings, some facilities were closed. For example, less than 40% of EmONC facilities surveyed in the DRC provided services on an around-the-clock basis (18). Infrastructure and supply availability were repeatedly mentioned as significant factors affecting a facility's ability to deliver ENC (15, 18, 20, 23). In Yemen, clinics and supply warehouses had been destroyed or damaged by air strikes, shelling, and looting (15). Sami, et al*.* reported that in South Sudan, although all clinics had running water, two of the five facilities surveyed had no electricity for at least half of each month (20). Irregularity of supply chains produced insufficient medical goods for service demands. A cross-sectional quality assessment of public health facilities in Afghanistan demonstrated that only 53.8% of facilities had blankets or towels available for thermal care; all facilities lacked at least one item needed for neonatal resuscitation (23). A functioning suction device and appropriate-sized face mask were available in less than 75% of the facilities surveyed. Other studies in Cameroon, Chad, Niger, Somalia, and South Sudan reported similar supply shortages of critical medical commodities (20, 21, 24).

In addition to lacking material goods, facilities did not have functional processes in place to be ready for ENC. The lack of institutional guidelines for neonatal resuscitation and infection prevention in some settings was particularly notable. In Afghanistan, for example, only 48% of public sector hospitals surveyed had a protocol for ENC, and 44.7% had guidelines for emergency obstetric and newborn care (23). Another study found that the lack of appropriate escalation guidelines for neonates who do not adequately respond to the Helping Babies Breathe resuscitation algorithm created circumstances in which “resuscitation [was] conducted in an ad hoc manner” (24). The studies included in this systematic review also revealed that facilities had inadequate referral mechanisms, health information systems, newborn registries, and adherence to quality-related process indicators (15, 18, 22, 24).

### Humanitarian crisis-related factors

Only one article from the Yemeni context described the contextual political, economic, and security barriers related to protracted humanitarian crises. There, two warring parties – the internationally recognized government and the de-facto authority – essentially acted as dueling coordinators of health system activities, leading to weakened authority, duplication of staff, confusion about service priorities, and erratic communication (15). In this study, Yemeni Ministry of Public Health and Population (MoPHP) officials reported feeling that their ability to manage programs and decide on health priorities was particularly hampered after the conflict escalated in 2015, a time when international agencies and donors became the principal decision-makers. MoPHP officials reported that external priorities, such as outbreak control and acute malnutrition care, drove resource allocation, whereas a fuller investment in health systems strengthening, infrastructure rebuilding, primary health care, and a wide array of reproductive, maternal, neonatal, child, and adolescent health services were not prioritized in all governorates (15). In this study, MoPHP respondents also reported the toll of insecurity on their work. Intimidation, injury, fear for family members, and acute and chronic stress were relevant barriers to MoPHP respondents' ability to carry out their work (15).

### Quality assessment

All studies met the criteria for screening using the STROBE checklist, and all were found to be high quality. The STROBE assessment did identify areas of low-quality reporting for each study, however (10). Only seven studies described efforts to address potential sources of bias in relation to study results (16, 18–23). Two studies lacked a detailed description of their participants (or non-participants) at each stage of the study or to justify the final sample size (16, 24).

## Discussion

The results of this systematic review reveal several obstacles to the delivery of ENC in humanitarian settings; our findings, described in Table 3, echo earlier studies that detail similar barriers. As geography may impose access constraints for patients, newborn care was noted to be less frequent in remote or out-of-camp settings (11, 25). Staff-related barriers were noted by many studies that cited high turnover, local insecurity, absenteeism, lack of female workers, and low salaries as critical factors that prevent retention (25–28). Having an inadequately trained cadre of staff is yet another barrier, as SBAs are not routinely present in conflict settings (25, 27, 29). Funding shortfalls have created facilities with poor infrastructure and insufficient newborn-specific supplies (25, 27). Health systems also face issues related to poor referral mechanisms, lack of standardized protocols, and inadequate health information systems (25, 26, 30). Barriers related to the overall ecosystem, such as political instability and the politicization of health, have been cited by other researchers, who note that even the most well-designed technical interventions can be imperiled in a humanitarian crisis (5, 6).

While the Field Guide provides several valuable clinical recommendations, it is critical to first identify high-impact, low-cost actions for all newborns that can potentially decrease mortality. The impact is potentially significant, as Bhutta, et al*.* estimated in 2014 that 420,000 lives could be saved by 2025 through improvements in ENC actions (31). Several studies have classified immediate ENC behaviors that fall into this category, such as the provision of thermal care and delayed bathing to prevent hypothermia, the promotion of exclusive breastfeeding within the first hour of life, vigorous stimulation of babies who are not spontaneously breathing, and the promotion of hygienic umbilical cord and skin care (31–33). Infection prevention measures at home and in a facility have also been demonstrated to decrease neonatal mortality (31). Other low-cost, promotive actions for healthy newborns include skin-to-skin contact, tetracycline ophthalmic ointment, intramuscular vitamin K prophylaxis, and weighing and registering the baby. Pre-discharge newborn examination and the concomitant provision of anticipatory guidance are simple and life-saving interventions that were not routinely performed in the records reviewed for this study (22–24). Many of these actions can occur at the community level and require minimal material or training inputs (33).

After considering which actions to target, it is important to consider how to best facilitate ENC delivery. Important patient-side facilitators of ENC include improving maternal knowledge of danger signs, so they know when to seek facility-based care (34, 35). Community-based educational initiatives, supplemented by education during antenatal visits, may improve maternal knowledge. Increased maternal education may promote facility-based births, which the Field Guide specifically encourages (7). A study in Darfur, for example, found that antenatal maternal health education was associated with a 43% reduction in home deliveries performed by TBAs (36). A community-based program that engaged with Rohingya mothers and community leaders in Bangladesh enabled an increase in facility-based birth and greater acceptance of delivery with skilled attendance and postnatal care with home visits (37). This study also provides an example in which some aspects of care were decentralized, through the employment of community midwives, in order to facilitate access.

Efforts to train and retain staff can improve ENC delivery in humanitarian settings. Trainings targeting high-impact ENC behaviors have the potential to decrease neonatal mortality through improvements in provider knowledge and attitudes (38). Trainings are beneficial when they are conducted in cross-functional teams, are complemented by mentorship, are linked to on-the-job observation and feedback mechanisms, and if refreshers occur regularly (15, 22, 23, 31). While the focus of this paper was ENC, it is important to note that trainings should also include interventions for small and ill babies, such as kangaroo mother care (KMC), timely antibiotic administration, and resuscitation (31, 39). Trainings may be complemented by shifting or sharing tasks when appropriate (26, 40). In addition to trainings, it is necessary to consider hiring locally and providing staff incentives, such as pay-for-performance and opportunities for advancement.

To be successful, trainings must also be accompanied by improvements at the facility and health system level. Infrastructure should be designed so mothers and babies can stay together for breastfeeding. Clinical protocols can help align care with high-quality standards while providing staff with just-in-time job support. Improvements in data collection at the facility level, including routine measurement of vital statistics, would not only allow for better measurement of service availability, coverage, and quality, but it would also allow for documenting needs and advocating for priorities (41). Facilities may also benefit from adopting neonatal signal functions related to essential and emergency care. These signal functions should be both aligned with service coverage targets from the Every Newborn Action Plan (ENAP), and adapted to humanitarian settings (3). They can be utilized in national, subnational, and facility-based assessments of service availability and coverage (42). Increasingly, these signal functions should be incorporated in assessments of quality of ENC, as outlined in guidelines WHO published in March 2022 (43). ENC is a central element of universal health coverage and must be incorporated into national and humanitarian packages of care.

The barriers and facilitators listed so far – related to patients, staff, and facilities – are shaped by the milieu in which they exist, namely the conflict settings themselves. While improvements in political stability and security would be conducive to the resumption of ENC services in humanitarian settings, “strategic” governance, or an approach in which the “minimal governance conditions required to implement” core ENC interventions may be sufficient, as has been demonstrated in several regions of instability and poor governance (6, 44). Other strategies, such as decentralization of operations and negotiated access to populations, also play a role in facilitating neonatal health interventions in humanitarian crises (40).

## Limitations

This study is not without its limitations. African countries are heavily represented in this study, and there is a relative dearth of other regions. Despite this bias toward Africa, we may have missed the perspectives of regional Arabic- and French-language scholars due to our English language limitation. We limited our focus to interventions that primarily addressed the neonate. In reality, as the Roadmap to Accelerate Progress for Every Newborn in Humanitarian Settings 2020–2024 emphasizes, mother-baby dyads are central to understanding the continuum of ENC, which begins antenatally (45). Our focus on newborn interventions may not have allowed us to capture the full range of barriers and facilitators to ENC delivery by not adequately considering pregnancy and parturition. We also did not focus on prematurity, low birth weight, intrapartum complications, or serious bacterial infections, which are significant causes of morbidity and mortality in the neonatal period. ENC services must be linked with interventions targeting small and ill babies. Next, while a few quasi-experimental studies were included, most articles were cross-sectional or retrospective, reflecting the logistical and ethical obstacles related to conducting research in an insecure environment. This is reinforced by the fact that only academic, peer-reviewed literature was included in this review. Operational research, often reported through grey literature and conducted by aid organizations in the field, was omitted. Finally, most studies reviewed here utilized health facility data and did not review community-based strategies or home births. There is likely a selection bias, as data about mothers and babies who could not access facilities are missing from this review. Indeed, while the Field Guide encourages facility-based births whenever possible, community-based strategies for home births are important to consider when thinking about ENC holistically.

## Conclusions

This systematic review outlines numerous barriers to ENC in humanitarian settings. Understanding barriers to ENC delivery will allow for the implementation of tailored strategies that can be aligned with international guidelines, such as ENAP, and adapted to humanitarian settings. Clarifying these barriers will not only strengthen service availability and coverage, but can also enable a renewed focus on improving the quality of newborn care in humanitarian settings.

## Data Availability

The original contributions presented in the study are included in the article and /**Supplementary Material**. further inquiries can be directed to the corresponding author.
