# Supplementary material for: The delivery of essential newborn care in conflict settings: A systematic review
Source: Front Pediatr. 2022 Nov 1;10:937751. doi: 10.3389/fped.2022.937751 (PMC9663655; doi:10.3389/fped.2022.937751)
Supplement: Supplementary file 1 [file Supplementary_Appendix_1.docx]

**Supplementary Appendix 1. Literature search syntax**

* Adapted from Munyuzangabo, et al. 2021 and Warren, et al. 2015.

**Conflict-related terms:**

1. disasters/ or emergencies/ or mass casualty incidents/ or disaster victims/
   1. 'disaster'/exp OR 'emergency'/exp OR 'mass disaster'/exp OR 'disaster victim'/exp
   2. (MH "Disasters+" OR MH "Natural Disasters+" OR MH "Mass Casualty Incidents")
2. ((disaster or disasters or catastrophe or catastrophes) adj5 (human or manmade or "man made")).tw,kf.
   1. ((disaster or disasters or catastrophe or catastrophes) NEAR/5 (human or manmade or ‘man made’)):ti,ab,kw
   2. ((disaster or disasters or catastrophe or catastrophes) n3 manmade) OR ((disaster or disasters or catastrophe or catastrophes) n3 “man made”) OR ((disaster or disasters or catastrophe or catastrophes) n3 human)
3. ("mass casualty" or "mass casualties" or "mass fatalities" or "mass fatality").tw,kf.
   1. ('mass casualty':ti,ab,kw OR 'mass casualties':ti,ab,kw OR 'mass fatalities':ti,ab,kw OR 'mass fatality':ti,ab,kw)
   2. TX ("mass casualty" or "mass casualties" or "mass fatalities" or "mass fatality)
4. "warfare and armed conflicts"/ or armed conflicts/ or warfare/ or biological warfare/ or bioterrorism/ or chemical warfare/ or chemical terrorism/ or nuclear warfare/ or psychological warfare/ or war crimes/ or ethnic cleansing/ or genocide/ or holocaust/ or war exposure/ or war-related injuries/
   1. ('military phenomena'/exp OR 'war'/exp OR 'warfare'/exp OR 'biological warfare'/exp OR 'bioterrorism'/exp OR 'chemical warfare'/exp OR 'chemical terrorism'/exp OR 'atomic warfare'/exp OR 'war crime'/exp OR 'ethnic cleansing'/exp OR 'genocide'/exp OR 'holocaust'/exp OR 'war exposure'/exp OR 'battle injury'/exp)
   2. (MH "War+" OR MH "War Crimes+" OR MH "Chemical Warfare" OR MH "Nuclear Warfare" OR MH "Holocaust+")
5. ((armed or zone or political or civil) adj3 (conflict or conflicts or attack or attacks or war or wars or "no fly")).tw,kf.
   1. ((armed OR zone OR political OR civil) NEAR/3 (conflict OR conflicts OR attack OR attacks OR war OR wars OR 'no fly')):ti,ab,kw
   2. (armed N3 (conflict OR conflicts OR attack OR attacks OR war OR wars OR 'no fly')) OR (zone N3 (conflict OR conflicts OR attack OR attacks OR war OR wars OR 'no fly')) OR (political N3 (conflict OR conflicts OR attack OR attacks OR war OR wars OR 'no fly')) OR (civil N3 (conflict OR conflicts OR attack OR attacks OR war OR wars OR 'no fly'))
6. ("war related injuries" or "war related traumas" or "war related injury" or "war related trauma").tw,kf.
   1. (‘war related injurie*’ OR ‘war related trauma*’):ti,ab,kw
   2. TX ("war related injuries" or "war related traumas" or "war related injury" or "war related trauma")
7. ("militant group" or "militant groups" or "militant organization" or "militant organizations" or "militant organisation" or "militant organisations").tw,kf.
   1. ('militant group*’ OR 'militant organization*' OR 'militant organisation*’):ti,ab,kw
   2. TX ("militant group" or "militant groups" or "militant organization" or "militant organizations" or "militant organisation" or "militant organisations")
8. ("biological terrorism" or bioterrorism or biowarfare or "chemical terrorism" or "ethnic cleansing" or "ethnic cleansings" or "gas poisoning" or genocide or holocaust or holocausts or "nuclear terrorism" or "war exposure" or "war exposures").tw,kf.
   1. ('biological terrorism' OR bioterrorism OR biowarfare OR 'chemical terrorism' OR 'ethnic cleansing' OR 'ethnic cleansings' OR 'gas poisoning' OR genocide OR holocaust OR holocausts OR 'nuclear terrorism' OR 'war exposure' OR 'war exposures'):ti,ab,kw
   2. TX ("biological terrorism" or bioterrorism or biowarfare or "chemical terrorism" or "ethnic cleansing" or "ethnic cleansings" or "gas poisoning" or genocide or holocaust or holocausts or "nuclear terrorism" or "war exposure" or "war exposures")
9. Disaster Medicine/
   1. (‘disaster medicine’/exp)
   2. (“disaster medicine)
10. ((emergency or emergencies) adj5 (environ* or human or manmade or "man made" or nature or natural or weather)).tw,kf.
    1. ((emergency OR emergencies) NEAR/5 (environ* OR human OR manmade OR 'man made' OR nature OR natural OR weather)):ti,ab,kw
11. Starvation/
    1. (‘starvation’/exp)
    2. (MH "Starvation")
12. (famine or famines or starvation or starvations).tw,kf.
    1. (famine* or starvation*):ti,ab,kw
    2. TX (famine* OR starvation*)
13. refugees/
    1. (‘refugee’/exp)
    2. (MH “refugees”)
14. (evacuee or evacuees or refugee or refugees or squatter or squatters or transients).tw,kf.
    1. (evacuee or evacuees or refugee or refugees or squatter or squatters or transients):ti,ab,kw
    2. TX (evacuee or evacuees or refugee or refugees or squatter or squatters or transients)
15. (humanitarian adj2 (aid or response or relief or crisis or crises or emergency or emergencies or disaster or disasters)).tw,kf.
    1. (humanitarian NEXT/2 (aid or response or relief or crisis or crises or emergency or emergencies or disaster or disasters)):ti,ab,kw
    2. (humanitarian N2 (aid or response or relief or crisis or crises or emergency or emergencies or disaster or disasters))
16. ("displaced children" or "displaced families" or "displaced family" or "displaced individuals" or "displaced internally" or "displaced men" or "displaced people" or "displaced peoples" or "displaced person" or "displaced persons" or "displaced population" or "displaced populations" or "displaced women" or "forced displacement" or "forced displacements" or "internal displaced" or "internal displacement" or "internally displaced" or "population displaced" or "population displacement").tw,kf.
    1. ('displaced children' OR 'displaced families' OR 'displaced family' OR 'displaced individuals' OR 'displaced internally' OR 'displaced men' OR 'displaced people' OR 'displaced peoples' OR 'displaced person' OR 'displaced persons' OR 'displaced population' OR 'displaced populations' OR 'displaced women' OR 'forced displacement' OR 'forced displacements' OR 'internal displaced' OR 'internal displacement' OR 'internally displaced' OR 'population displaced' OR 'population displacement'):ti,ab,kw
    2. (TX ("displaced children" or "displaced families" or "displaced family" or "displaced individuals" or "displaced internally" or "displaced men" or "displaced people" or "displaced peoples" or "displaced person" or "displaced persons" or "displaced population" or "displaced populations" or "displaced women" or "forced displacement" or "forced displacements" or "internal displaced" or "internal displacement" or "internally displaced" or "population displaced" or "population displacement"))
17. (((camp or camps) and displac*) or "protected village*").tw,kf.
    1. ((camp* AND displac*) OR 'protected village*’):ti,ab,kw
    2. ((camp* AND displac*) OR “protected village*”)
18. (crisis adj5 (environ* or human or manmade or "man made”)).tw,kf.
    1. (crisis NEAR/5 (environ* or human or manmade or 'man made')):ti,ab,kw
    2. (crisis N5 (environ* OR human OR manmade OR “man made”))
19. (crises adj5 (environ* or human or manmade or "man made”)).tw,kf.
    1. (crises NEAR/5 (environ* or human or manmade or 'man made')):ti,ab,kw
    2. (crises N5 (environ* OR human OR manmade OR “man made”))

**Population of interest:**

1. child/ or child, preschool/ or infant/ or infant, newborn/ or infant, low birth weight/ or infant, small for gestational age/ or infant, very low birth weight/ or infant, extremely low birth weight/ or infant, postmature/ or infant, premature/ or infant, extremely premature/
   1. 'child'/exp OR 'preschool child'/exp OR 'infant'/exp OR 'newborn'/exp OR 'high risk infant'/exp OR 'newborn'/exp OR 'low birth weight'/exp OR 'small for date infant'/exp OR 'very low birth weight'/exp OR 'extremely low birth weight'/exp OR 'prematurity'/exp OR 'postmaturity'/exp
   2. (MH "Infant, Premature" OR MH "Infant, Postmature" OR MH "Infant, Very Low Birth Weight" OR MH "Infant, Small for Gestational Age" OR MH "Infant, Low Birth Weight+" OR MH "Infant, Large for Gestational Age" OR MH "Infant, Newborn+" OR MH "Infant+" OR MH "Child+")
2. (infan* or newborn* or "new born*" or neonat* or baby* or babies or toddler* or boy or boys or boyhood or girl* or kid or kids or child* or pediatric* or paediatric* or peadiatric* or prematur* or preterm*).mp. or school*.tw.
   1. (infan* or newborn* or "new born*" or neonat* or baby* or babies or toddler* or boy or boys or boyhood or girl* or kid or kids or child* or pediatric* or paediatric* or peadiatric* or prematur* or preterm*):ti,ab,kw
   2. TX (infan* or newborn* or "new born*" or neonat* or baby* or babies or toddler* or boy or boys or boyhood or girl* or kid or kids or child* or pediatric* or paediatric* or peadiatric* or prematur* or preterm*)

**Neonatal care-related terms:**

1. Perinatal Care/
   1. ‘perinatal care’/exp
   2. (MH "Perinatal Care")
2. ("perinatal care" or "peri natal care" or "perinatal care" or "peri natal care").tw,kf.
   1. ('perinatal care' OR 'peri natal care'):ti,ab,kw
   2. (TX “perinatal care” OR “peri natal care))
3. Peripartum Period/
   1. (‘perinatal period’/exp OR ‘peripartum period’)
4. ("peripartum* period*" or "perinatal* period*" or "peri natal* period*").tw,kf.
   1. (‘peripartum period’ OR ‘perinatal period’ OR ‘peri natal period’):ti,ab,kw
   2. (TX (“peripartum period” OR “perinatal period” OR “peri natal period”)
5. Parturition/
   1. (‘birth’/exp)
   2. (MH “childbirth+”)
6. (birth or childbirth* or parturition* or "safe delivery" or "safely delivered").tw,kf.
   1. (‘birth’ OR ‘childbirth’ OR ‘parturition’ OR ‘safe delivery’ OR ‘safely delivered’):ti,ab,kw
   2. (TX (birth OR childbirth OR parturition OF “safe delivery” OR “safely delivered”))
7. Stillbirth/
   1. (‘stillbirth’/exp)
   2. (MH “Perinatal death”)
8. (stillbirth or stillbirths or stillborn or stillborns or "still birth" or "still births" or "still born" or "still borns").tw,kf.
   1. (‘stillbirth*’ OR ‘stillborn*’ OR ‘still birth*’ OR ‘still born*’):ti,ab,kw
   2. (TX (stillbirth or stillbirths or stillborn or stillborns or "still birth" or "still births" or "still born" or "still borns"))
9. (emoc or emonc or cemoc or bemoc or cemonc or bemonc).tw,kf.
   1. (emoc or emonc or cemoc or bemoc or cemonc or bemonc):ti,ab,kw
   2. (TX (emoc or emonc or cemoc or bemoc or cemonc or bemonc))
10. Postnatal Care/
    1. (‘postnatal care’/exp)
    2. (MH “postnatal care”)
11. Postpartum Period/
    1. (‘puerperium’/exp)
    2. (MH “postnatal period+”)
12. (postnatal or "post natal" or postpartum or "post partum" or puerperium or puerperal).tw,kf.
    1. (postnatal OR ‘post natal’ OR postpartum OR ‘post partum’ OR puerperium OR puerperal):ti,ab,kw
    2. (TX (postnatal or "post natal" or postpartum or "post partum" or puerperium or puerperal))
13. ("neonatal health" or "newborn health" or "new born health" or "infant health").tw,kf.
    1. (‘newborn health’ OR ‘newborn healthcare’ OR ‘new born health’ OR ‘infant health’):ti,ab,kw
    2. (TX (“neonatal health" or "newborn health" or "new born health" or "infant health"))
14. Resuscitation/ and exp Infant, Newborn/
    1. (‘resuscitation’/exp AND ‘newborn’/exp)
    2. (MH “resuscitation+” AND MH “infant, newborn+”)
15. ((resuscitat* or reanimat*) adj3 (neonat* or newborn* or "new born*")).tw,kf.
    1. ((resuscitat* OR reanimate*) NEAR/3 (neonat* OR newborn* OR ‘new born’)):ti,ab,kw
    2. ((TX resuscit* N3 (neonat* OR newborn* OR “new born*)) OR (TX reanimate* N3 (neonat* OR newborn* OR “new born))
16. Infant Mortality/ and exp Infant, Newborn/
    1. ('infant mortality'/exp AND ‘newborn’/exp)
    2. (MH “infant mortality” AND MH “infant, newborn+”)
17. Perinatal Mortality/
    1. (‘perinatal mortality’/exp)
18. ("neonat* mortalities" or "neonat* mortality" or "neonatal survival" or "newborn mortalities" or "newborn mortality" or "new born mortalities" or "new born mortality" or "newborn survival" or "new born survival" or "perinatal death rate" or "perinatal mortalities" or "perinatal mortality" or "postneonat* mortality" or "postneonat* mortalities").tw,kf.
    1. ('neonat* mortalities' or 'neonat* mortality' or 'neonatal survival' or 'newborn mortalities' or 'newborn mortality' or 'new born mortalities' or 'new born mortality' or 'newborn survival' or 'new born survival' or 'perinatal death rate' or 'perinatal mortalities' or 'perinatal mortality' or 'postneonat* mortality' or 'postneonat* mortalities'):ti,ab,kw
    2. (TX ("neonat* mortalities" or "neonat* mortality" or "neonatal survival" or "newborn mortalities" or "newborn mortality" or "new born mortalities" or "new born mortality" or "newborn survival" or "new born survival" or "perinatal death rate" or "perinatal mortalities" or "perinatal mortality" or "postneonat* mortality" or "postneonat* mortalities"))
19. Hypoxia/ and exp Infant, Newborn/
    1. (‘hypoxia’/exp AND ‘newborn’/exp)
20. Asphyxia Neonatorum/
    1. (‘newborn hypoxia’/exp)
    2. (MH “hypoxia-ischemia, brain, neonatal”)
21. ("asphyxia neonatorum" or "birth asphyxia" or "neonatal anoxia" or "neonatal asphyxia" or "neonatal hypoxia" or "neonate asphyxia" or "neonatus hypoxia" or "new born asphyxia" or "newborn asphyxia").tw,kf.
    1. ('asphyxia neonatorum' or 'birth asphyxia' or 'neonatal anoxia' or 'neonatal asphyxia' or 'neonatal hypoxia' or 'neonate asphyxia' or 'neonatus hypoxia' or 'new born asphyxia' or 'newborn asphyxia’):ti,ab,kw
    2. (TX (“asphyxia neonatorum" or "birth asphyxia" or "neonatal anoxia" or "neonatal asphyxia" or "neonatal hypoxia" or "neonate asphyxia" or "neonatus hypoxia" or "new born asphyxia" or "newborn asphyxia"))
22. Neonatal Sepsis/
    1. (‘newborn sepsis’/exp)
    2. (MH “neonatal sepsis”)
23. ("neonatal early onset sepses" or "neonatal early onset sepsis" or "neonatal late onset sepses" or "neonatal late onset sepsis" or "neonatal sepses" or "neonatal sepsis" or "neonatal septicaemia" or "neonatal septicemia" or "new born sepses" or "new born sepsis" or "new born septicaemia" or "new born septicemia" or "newborn sepses" or "newborn sepsis" or "newborn septicaemia" or "newborn septicemia").tw,kf.
    1. ('neonatal early onset sepses' or 'neonatal early onset sepsis' or 'neonatal late onset sepses' or 'neonatal late onset sepsis' or 'neonatal sepses' or 'neonatal sepsis' or 'neonatal septicaemia' or 'neonatal septicemia' or 'new born sepses' or 'new born sepsis' or 'new born septicaemia' or 'new born septicemia' or 'newborn sepses' or 'newborn sepsis' or 'newborn septicaemia' or 'newborn septicemia’):ti,ab,kw
    2. (TX ("neonatal early onset sepses" or "neonatal early onset sepsis" or "neonatal late onset sepses" or "neonatal late onset sepsis" or "neonatal sepses" or "neonatal sepsis" or "neonatal septicaemia" or "neonatal septicemia" or "new born sepses" or "new born sepsis" or "new born septicaemia" or "new born septicemia" or "newborn sepses" or "newborn sepsis" or "newborn septicaemia" or "newborn septicemia"))
24. Kangaroo-Mother Care Method/
    1. (‘kangaroo care’/exp)
    2. (MH “kangaroo care”)
25. ("kangaroo mother care" or "kangaroo mother method" or "skin to skin").tw,kf.
    1. (‘kangaroo mother care’ OR ‘kangaroo mother method’ OR ‘skin to skin’):ti,ab,kw
    2. (TX (“kangaroo mother care" or "kangaroo mother method" or "skin to skin”))
26. exp meningitis/ and exp Infant, Newborn/
    1. (‘meningitis’/exp AND ‘newborn’/exp)
    2. (MH “meningitis+”) AND MH “infant, newborn+”)
27. ((meningitides or meningitis or "meningeal inflammation" or pachymeningitides or pachymeningitis or "perimeningeal infections") adj3 (neonat* or newborn * or "new born*")).tw,kf.
    1. ((meningitides or meningitis or ‘meningeal inflammation’ or pachymeningitides or pachymeningitis or ‘perimeningeal infections’) NEAR/3 (neonat* or newborn* or ‘new born*’)):ti,ab,kw
    2. ((mening* N3 (neonat* OR newborn OR “new born”)) OR (pachymening* N3 (neonat* OR newborn OR “new born”)) OR (perimeningeal N4 (neonat* OR newborn OR “new born*)))
28. ("bronze baby syndrome" or erythroleukoblastosis or ((jaundice or icterus) adj3 (neonat* or newborn * or "new born*"))).tw,kf.
    1. ((((jaundice OR icterus) NEAR/3 (neonat* OR newborn OR 'new born')):ti,ab,kw) OR 'bronze baby syndrome':ti,ab,kw OR erytholeukoblastosis:ti,ab,kw)
    2. ((jaundice N3 (neonat* OR newborn OR “new born*”)) OR (icterus N3 (neonat OR newborn OR “new born”)) OR “bronze baby syndrome” OR erythroleukoblastosis)
29. Fetal Growth Retardation/
    1. ('intrauterine growth retardation'/exp)
    2. (MH “fetal growth retardation”)
30. ("congenital hypotrophy" or "fetal growth disorder" or "fetal growth restriction" or "fetal growth retardation" or "fetus growth disorder" or "fetus growth retardation" or "foetal growth restriction" or "foetal growth retardation" or "growth retardation in utero" or "in utero growth retardation" or "intrauterine growth restriction" or "intrauterine growth retardation" or "iugr" or "prenatal growth retardation" or "retarded intrauterine growth").tw,kf.
    1. ('congenital hypotrophy' or 'fetal growth disorder' or 'fetal growth restriction' or 'fetal growth retardation' or 'fetus growth disorder' or 'fetus growth retardation' or 'foetal growth restriction' or 'foetal growth retardation' or 'growth retardation in utero' or 'in utero growth retardation' or 'intrauterine growth restriction' or 'intrauterine growth retardation' or 'iugr' or 'prenatal growth retardation' or 'retarded intrauterine growth’):ti,ab,kw
    2. (TX ("congenital hypotrophy" or "fetal growth disorder" or "fetal growth restriction" or "fetal growth retardation" or "fetus growth disorder" or "fetus growth retardation" or "foetal growth restriction" or "foetal growth retardation" or "growth retardation in utero" or "in utero growth retardation" or "intrauterine growth restriction" or "intrauterine growth retardation" or "iugr" or "prenatal growth retardation" or "retarded intrauterine growth”))
31. ("neonatal care" or "newborn care" or "new born care" or “essential neonatal care” or “essential newborn care” or “essential new born care” or "helping babies breathe" or "helping babies survive").tw,kf.
    1. (‘neonatal care’ OR ‘newborn care’ OR ‘new born care’ OR ‘essential neonatal care’ OR ‘essential newborn care’ OR ‘essential new born care’ OR ‘helping babies breath’ OR ‘helping babies survive’):ti,ab,kw
    2. (TX (“neonatal care" or "newborn care" or "new born care" or “essential neonatal care” or “essential newborn care” or “essential new born care” or "helping babies breathe" or "helping babies survive"))
32. Jaundice, Neonatal
    1. (‘newborn jaundice’/exp)
    2. (MH “jaundice, neonatal”)

**General approach:** Must include any conflict-related term, any population of interest term, **and** any neonatal care term.
